# Secular trends in the mortality of gastrointestinal cancers across China, Japan, the US, and India: An age-period-cohort, Joinpoint analyses, and Holt forecasts

**DOI:** 10.3389/fpubh.2022.925011

**Published:** 2022-09-29

**Authors:** Yiran Cui, Gang Cheng, Gang Tian, Simin He, Yan Yan

**Affiliations:** Department of Epidemiology and Medical Statistics, Xiangya School of Public Health, Central South University, Changsha, China

**Keywords:** colon cancer, esophageal cancer, stomach cancer, age-period-cohort analysis, global burden

## Abstract

**Background:**

Colon cancer, esophageal cancer, and stomach cancer are the common causes of morbidity and mortality in China, Japan, the US., and India. The current study aims to assess and compare secular trends of the mortality of gastrointestinal cancers during the period, 1990–2017 in age-specific, time period, and birth cohort effects.

**Method:**

We used the Joinpoint model to collect age-standardized mortality rates (ASMRs) for four countries. We designed an age-period-cohort (APC) analysis to estimate the independent effects on the mortality of three types of cancers.

**Result:**

The Joinpoint model shows that in addition to the death rate of esophageal cancer in Japan, the ASMR of esophageal cancer and stomach cancer in other countries declined rapidly. The APC analysis presented a similar pattern of age effect between four countries for colon cancer and stomach cancer, which increased from 20 to 89 age groups. Differently, the period effect rapidly increased for esophageal cancer and stomach cancer in the US, and the period effect in China presented a declining volatility, showing its highest value in 2007. In future, highest mortality trends are likely to occur in China.

**Conclusion:**

Therefore, the obvious increase in colon cancer recommended that earlier tactics must be performed to reduce mortality from specific causes from 2018 to 2027.

## Introduction

Colon cancer, esophageal cancer, and stomach cancer are very common gastrointestinal cancers (1). Not only these cancers remain as one of the most common malignancies, but they are often identified as leading causes of cancer death other than lung cancer (2). The longitudinal trends of these three cancers may be affected by many risk factors, such as dietary structure, lifestyle changes, and the implementation of screening (3). The monitoring data for colorectal cancer mortality rate was reported to be 6.9/100,000 in 2017 (4). Meanwhile, according to the data from global burden of disease (GBD), the disability-adjusted life years (DALYs) in China caused by colon cancer in 2017 were 4.25 million per year (doubled from 1990), accounting for 22.4% of the global burden of colon cancer in 2017 (4). The value of DALYs in China is significantly higher than that of the other three countries. In 2017, the DALYs value of colon cancer in Japan, the US, and India were 1.04, 1.68 and 1.81 million per year, respectively (5).

For esophageal cancer, although China's population accounts for nearly 20% of the world's population, it accounts for half of the world's total deaths from esophageal cancer (6). Esophageal cancer is the fourth most common cancer in China (7). According to the latest data from China, the age-standardized death rate of esophageal cancer is 8.36 per 100,000 cases, which is 1.52 times greater than the global rate (8). However, in Japan and the US, there is an interaction between genetic and environmental factors. It may be that the risk factors of esophageal cancer are related to lack of nutrition, unhealthy lifestyle, and smoking. However, the exact mechanism of esophageal cancer is still unclear (9). Among cancers caused by alcohol, the esophageal cancer accounts for the highest proportion in Japan. GBD2010 estimates the death of esophageal cancer caused by alcohol of 76,700 and 1,825 000 DALYs lost (10). The esophageal cancer is relatively rare in most Western Europe and North America, but overall, it is the sixth leading cause of death from cancer in the world (11). In India, the incidence of esophageal cancer may increase, and this increase is mainly found in people with lower socioeconomic class (12).

Stomach cancer occurs as a malignant tumor, and deaths due to stomach cancer in China account for about 50% of the global stomach cancer deaths (13). It is estimated that, about 498,000 people in China died of stomach cancer and about 1,364 people died every day in 2015. Stomach cancer has always been one of the main causes of death among the Chinese people (14). It is not only related to industrialization and urbanization of China, but also related to high-risk factors, such as Helicobacter pylori and unhealthy lifestyles (15). The incidence of stomach cancer showed significant geographical differences. Among them, Japan, China, Eastern Europe, and certain countries in Latin America are high-risk regions. Regions, such as the US and India are low-risk regions. Most of the world's gastric cancer cases occurs in developing countries, with East Asia having one of the highest mortality rates in the world, but the mortality rate from stomach cancer in East Asia has been declining (16).

## Methods

### Source of data

The age-standardized death and population data used in this study are obtained from the Institute for Health Metrics and Evaluation (IHME, http://ghdx.healthdata.org/). Global Health Data Exchange (GHDx) released by IHME provides a systematic assessment of important health issues and disease burdens in 197 countries and regions, providing a scientific basis for the formulation of relevant health policies (17). The data from IHME are obtained from surveys, censuses, vital statistics, and other health-related data collected from various countries around the world. Meanwhile, it is also an open database with worldwide credibility. The GBD not only estimates the incidence, prevalence, mortality, years of life lost (YLL), years lived with disability (YLD), and DALY indicators of each disease and injury, but also reports the year, location, age group, and sex. The purpose of GBD research is to establish comprehensive and comparable global health indicators.

### Statistical analysis

#### Age-period-cohort model

The mortality of colon cancer, esophageal cancer, and stomach cancer stratified by countries (China/Japan/the US/India), gender (male/female), and age groups (20–89 by 5 years) were calculated. These death rates were smoothed with 5-year moving averages to reveal secular trends. For the age-period-cohort (APC) analysis, the present study collected the data for the colon cancer, esophageal cancer, and stomach cancer din successive five-year periods from 1992–1997 to 2012–2017, and 14 five-year age groups, ranging from 20–24 years to 85-89 years, and 10 five-year birth cohorts, ranging from 1903–1907 to 1993–1997.

The APC model can simultaneously evaluate the contribution of age effect, period effect, and the corresponding birth effect on morbidity and mortality. It represents an epidemiological method, which can be used to extract information about historical changes in mortality and morbidity risks from cross-sectional data; it is also widely used in the fields of sociology, demography, and epidemiology.

As cohort = period - age, there is a multicollinearity between variables. Multicollinearity is a common problem in the application of APC models. Yang (2008) first proposed the intrinsic estimator (IE) to solve the multicollinearity problem in the application of the APC model (18–20). In this study, the model is expressed as follows:


(1) $$\begin{array}{l} \text{Y}=\text{log}\left(M\right)=\mu +\alpha age_{i}+\beta period_{j}+\gamma cohort_{k}+\;\varepsilon . \end{array}$$

*M* is defined as the death rate; α, β, and γ refer to three coefficients (α refers to the age effect, β represents the period effect, and γ is the cohort effect, μ and ε are defined as the intercept and random error, respectively. The IE method expresses the APC model in the following equation:


(2) $$\begin{array}{l} Y=Xb\;+\;\varepsilon , \end{array}$$

where *Y* represents the logarithm of gastrointestinal cancers mortality by age, period, and birth cohort; *X* represents the design matrix; *b* is a vector of age, period, and cohort-effect coefficients.

The dimension of matrix *X* is full rank minus one due to multicollinearity issues with age, period, and birth population. Therefore, the solution set *P* can be expressed as the direct sum of *N* and Θ:


(3) $$\begin{array}{l} \text{P}=N⊕\Theta , \end{array}$$

where the one-dimensional solution set *N* can be written as {*tB*_0_} and *X*·*tB*_0_=0. Obviously *B*_0_ is completely determined by the design matrix *X*. For any solution, *bˆ* of the APC model can be written as follows:


(4) $$\begin{array}{l} bˆ=B+\text{t}B_{0}, \end{array}$$

where *B* is the only definite solution of the APC model, that is, the IE solution of the APC model.

The APC analysis was performed using Stata 12.0 software. Moreover, the STATA software needs to install the APC package to implement APC model analysis. The APC package includes generalized linear model methods and IE methods. In the APC model, the effect of the period (or cohort) rate ratio (RR) represents the ratio of the age-specific values to the reference ratio in each period (or cohort). In addition, we considered the result *p* < 0.05 as statistically significant. We used Akaike information criteria (AIC) and Bayes Information Criteria (BIC) to evaluate the degree of model fitting.

#### Joinpoint regression analysis

Research on trend changes is an important issue for analyzing cancer mortality and morbidity data. We used Joinpoint to estimate the time trends of the age-standardized mortality rate (ASMR) of three cancers, and determined the changes in China, Japan, the US, and India in different years based on the turning points, and estimated the annual percentage change (APC), average annual percentage change (AAPC), and 95% of each trend. The model uses the standardized rate of cancer as the dependent variable and the year as the independent variable to establish a log-linear model. Gender and age group are secondary variables. In the model, the ratios are log-transformed and the standard errors are approximated by the binomial. In this model, the change of mortality in different years in these four countries was determined by breakpoints, and the temporal trend of gastrointestinal cancer mortality burden was more intuitively described. Monte Carlo permutation was used to test for significant changes at time points. APC and AAPC were used to characterize the changing trend of ASMR of gastrointestinal cancers. APC/AAPC > 0 indicates that the interval increases year by year, and APC/AAPC < 0 indicates that the interval decreases year by year. The model is implemented using the Joinpoint regression program version 4.7.0.0 of the Statistical Research and Application Department of the National Cancer Institute's Monitoring Research Program.

#### Fitting Holt exponential smoothing model

Holt model uses double exponential smoothing (21, 22). Two smoothing components are adjusted according to the level and trend of the data. Holt forecasting identifies the degree of dissimilarity in the development trend among system factors and generates and processes the original data to find the law of system changes, generates a data sequence with strong regularity, and then establishes the corresponding differential equation. This model aimed to predict the future development trend of things. The Holt forecast GM (1, 1) prediction model was established by Matlab 7.1 software. Holt model used in this study is mostly used in trend forecasting applications with a small number of time series. It is used to predict the death of colon cancer, esophageal cancer, and stomach cancer in China, Japan, the US, and India from 2018 to 2027.

## Results

### Descriptive analysis of death rate trends

**Figure 1:**
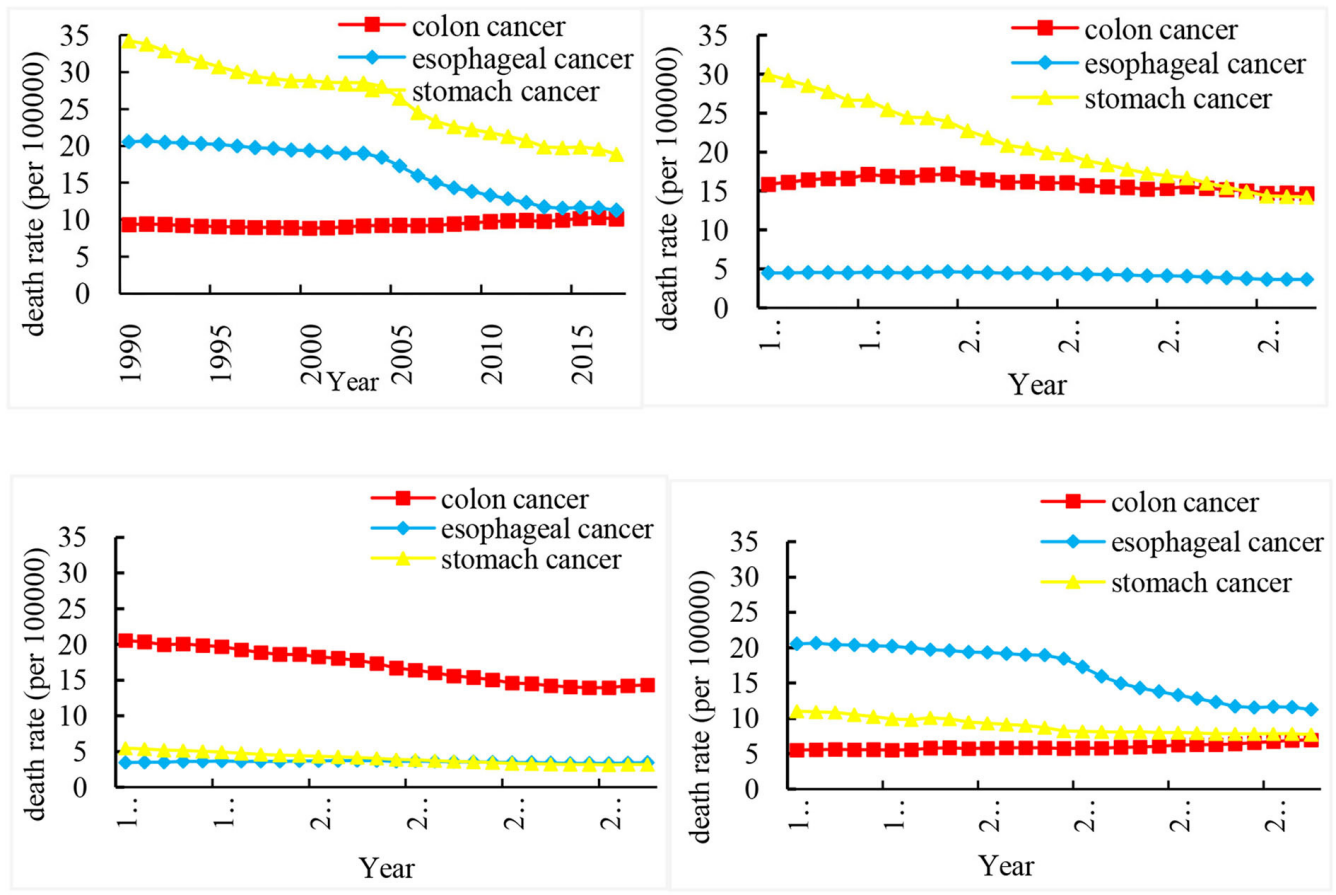
Trends in the age-standardized rates of three cancer deaths in China, Japan, US, and India from 1990 to 2017.

Figure 1 shows the ASMR of three cancers in China, Japan, the US, and India from 1990 to 2017. In China, the ASMR of stomach cancer is particularly significantly higher than that of colon cancer. In 2017, the death rate of colon cancer in China was very close to that of the esophageal cancer. However, the ASMR is the lowest in esophageal cancer in Japan, compared with stomach cancer and colon cancer. Besides, the trend of ASMR in the US is opposite to that in China, and stomach cancer has a lower death rate. The ASMR of colon cancer in the US is very high, and it has dropped from 20.61 per 100,000 in 1990 to 14.35 per 100,000 in 2017. The ASMR of esophageal cancer is almost at a higher level in India, higher than that of stomach cancer and colon cancer.

Table 1 shows the annual percentage change (APC) and average annual percent change (AAPC) of three cancer deaths in China, Japan, US, and India from 1990 to 2017. Joinpoint model showed that the death rate of esophageal cancer and stomach cancer decreased rapidly from 2004 to 2007 This trend can be seen for stomach cancer, with overall AAPC values of 2.2% (−2.5, −2.0) in China, −2.8% (−3.1, −2.5) in Japan, −2.0% (−2.2, −1.8) in the US, and −1.4% (−1.7, −1.0) in India.

**Table 1:** Trends in age-standardized death rates of colon, esophageal and stomach cancer in China, Japan, the U.S., and India, 1990–2017.

**Joinpoint model**	**Colon cancer**		**Esophageal cancer**		**Stomach cancer**	
	**Year**	APC^*^(95%CI)	**Year**	APC^*^(95% CI)	**Year**	APC^*^(95% CI)
**China**						
Trend 1	1990–1999	−0.6^*^ (−1.0, −0.2)	1990–2004	−0.8^*^ (−0.9, −0.7)	1990–1997	−2.3^*^ (−2.7, −1.8)
Trend 2	1999–2007	0.5^*^ (0.2, 0.8)	2004–2007	−7.2^*^ (−8.1, −6.2)	1997–2004	−0.6^*^ (−1.0, −0.2)
Trend 3	2007–2010	1.6^*^ (−0.2, 3.5)	2007–2013	−3.8^*^ (−4.1, −3.6)	2004–2007	−6.4^*^ (−8.1, −4.6)
Trend 4	2010–2017	0.7^*^ (0.4, 1.1)	2013–2017	−0.9^*^ (−1.5, −0.3)	2007–2017	−2.1^*^ (−2.3, −1.9)
AAPC^*^	1990–2017	0.3^*^ (0.1, 0.6)	1990–2017	−2.2^*^ (−2.4, −2.1)	1990–2017	−2.2^*^ (−2.5, −2.0)
**Japan**						
Trend 1	1990–1998	0.8^*^ (0.5, 1.1)	1995–1998	1.3 (−1, 4, 4.1)	1990–1996	−2.8^*^ (−3.2, −2.4)
Trend 2	1998–2008	−1.1^*^ (−1.3, −0.8)	1998–2001	4.3^*^ (1.6, 7.1)	1996–1999	−2.2 (−4.2, −0.1)
Trend 3	2008–2011	0.2 (−2.0, 2.6)	2001–2009	1.3^*^ (0.9, 1.6)	1999–2002	−4.0^*^ (−5.9, −2.0)
Trend 4	2011–2017	−1.0^*^ (−1.8, −0.1)	2009–2017	−0.5^*^ (−0.8, −0.1)	2002–2017	−2.7^*^ (−2.8, −2.6)
AAPC^*^	1990–2017	−0.4^*^ (−0.7, −0.1)	1990–2017	1.5^*^ (1.1, 1.9)	1990–2017	−2.8^*^ (−3.1, −2.5)
**The U.S**.						
Trend 1	1990–2001	−1.3^*^ (−1.4, −1.1)	1990–2002	0.6^*^ (0.4, 0.7)	1990–2002	−2.3^*^ (−2.5, −2.2)
Trend 2	2001–2012	−2.2^*^ (−2.4, −2.1)	2002–2014	−1.0^*^ (−1.1, −0.8)	2002–2012	−2.5^*^ (−2.7, −2.3)
Trend 3	2012–2017	0.0 (−0.9, 0.9)	2014–2017	0.9 (−1.4, 3.3)	2012–2017	−0.3 (−1.2, 0.6)
AAPC^*^	1990–2017	−1.4 (−1.6, −1.2)	1990–2017	−0.1 (−0.3, 0.2)	1990–2017	−2.0^*^ (−2.2, −1.8)
**India**						
Trend 1	1990–2002	0.5^*^ (0.3, 0.8)	1990–1995	−0.9^*^ (−1.8, −0.1)	1990–1995	−2.3^*^ (−3.0, −1.6)
Trend 2	2002–2005	−0.7 (−3.0, 1.6)	1995–1998	1.4 (−2.1, 5.0)	1995–1998	−0.2 (−3.1, 2.8)
Trend 3	2005–2013	1.4^*^ (1.1, 1.6)	1998–2006	−2.2^*^ (−2.6, −1.7)	1998–2005	−2.8^*^ (−3.2, −2.3)
Trend 4	2013–2017	2.2^*^ (1.5, 3.0)	2006–2017	0.5^*^ (0.2, 0.7)	2005–2017	−0.4^*^ (−0.6, −0.3)
AAPC^*^	1990–2017	0.9^*^ (0.6, 1.2)	1990–2017	−0.5^*^ (−0.9, −0.1)	1990–2017	−1.4^*^ (−1.7, −1.0)

* APC, annual percentage change; AAPC, average annual percent change; CI, confidence interval; *Significantly different from 0 at alpha = 0.05 (*p* < 0.05).

### The age, period, and cohort effects on colon cancer mortality

**Figure 2:**
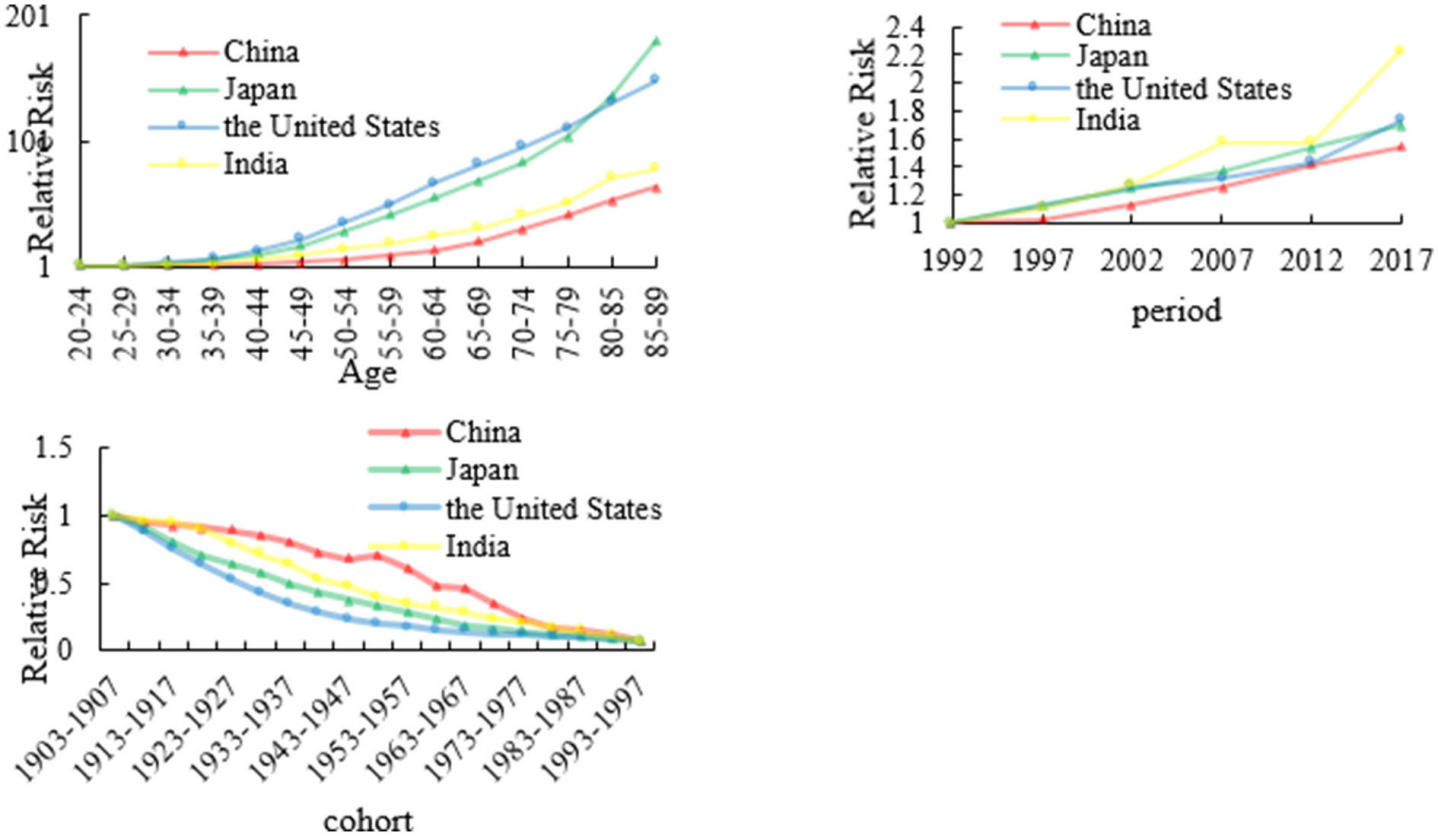
The age, period, and cohort effects on colon cancer mortality across four countries.

We calculated colon cancer RRs of four countries based on age, period, and cohort, which are shown in Figure 2 and Appendix 1. Regarding the age effect of colon cancer mortality, we set the age RR of colon cancer mortality in the 20–24 age group as 1. The RR values of China, Japan, the US, and India were 63.92, 180.17, 149.19, and 79.07, respectively. The RR values of the death rate of colon cancer in the four countries all increased with age. But generally speaking, the RR values of colon cancer mortality in Japan and the US are higher than those of China and India in all age groups.

For the period RR of colon cancer mortality, we set the RR to 1 of the colon cancer mortality in 1992. In 2017, the RR values of China, Japan, US, and India were 1.54, 1.70, 1.73, and 2.23, respectively. From 1992 to 2017, the RR value of death rate in the four countries showed an overall upward trend, while the RR of colon cancer mortality in India showed a tortuous upward trend. For the cohort effect, the RRs of colon cancer death rate in the four countries showed a downward trend with the cohort, and China had a slight upward trend in 1948–1952.

### The age, period, and cohort effects on esophageal mortality

**Figure 3:**
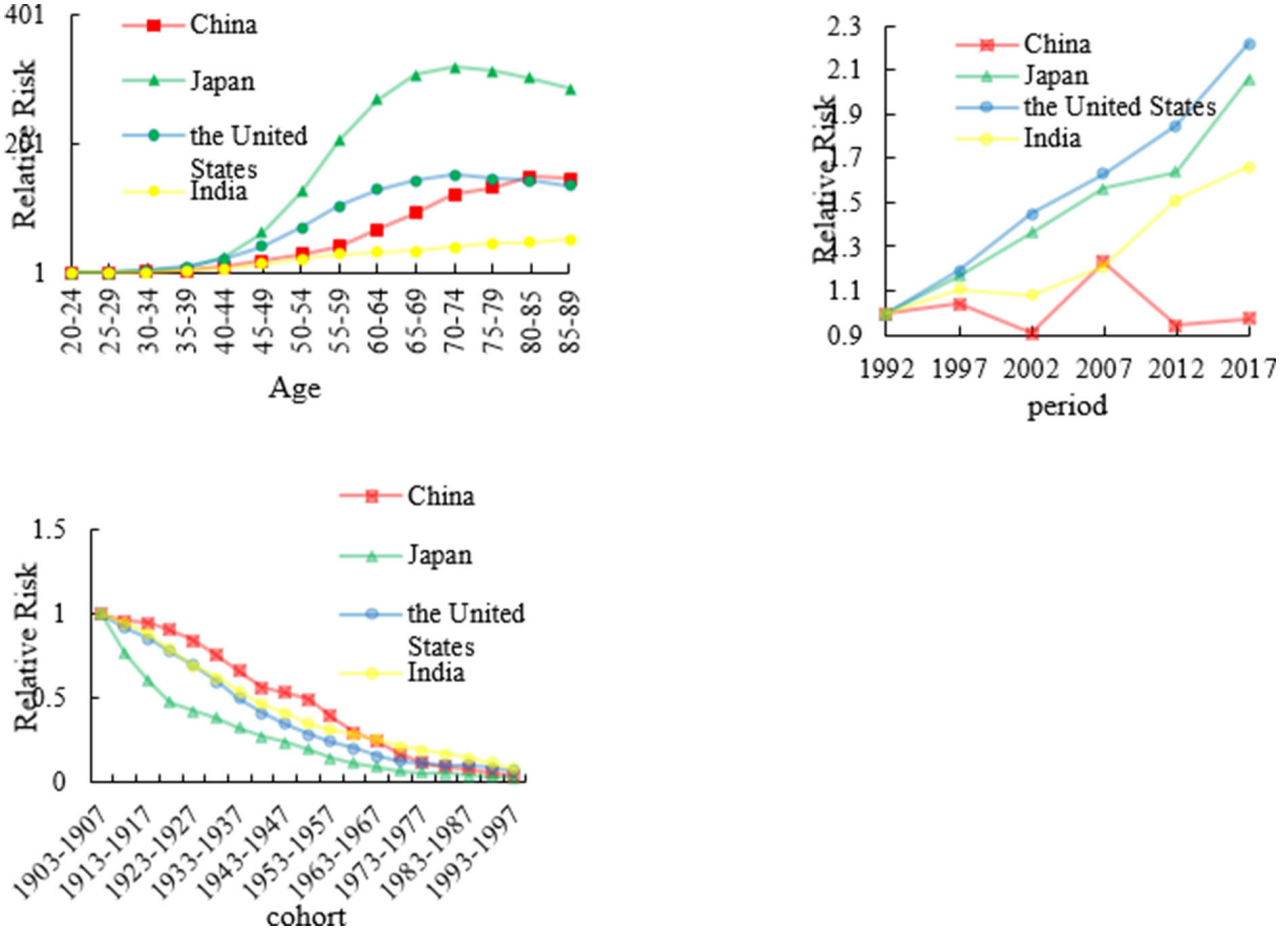
The age, period, and cohort effects on esophageal cancer mortality across four countries.

We analyzed esophageal RRs in China, Japan, the US, and India from 1992 to 2017 which are shown in Figure 3 and Appendix 2. The age effect in Japan increased approximately linearly with increasing age from 30 to 75 years and peaking at approximately 75 years. After 75 years of age, the age effect continued to decrease. The RR of the US has the similar trend as that of Japan. The age RR of other two countries increased all the time from 20 to 90 years age group. The period effect in China decreased from 1997 to 2002, and thereafter generally increased until the year 2007. After 2008, the period effect decreased again. However, the period RRs in Japan, US, and India has an upward trend from 1992 to 2017.

The cohort effect in China, Japan, the US, and India generally decreased from the 1910s to the 1990s. The cohort effect maintained a relatively stable trend before 1937 in China, Japan, and the US, which was followed by a decline. However, the cohort effect declined at an accelerated rate since the 1910s in Japan.

### The age, period, and cohort effects on stomach mortality

**Figure 4:**
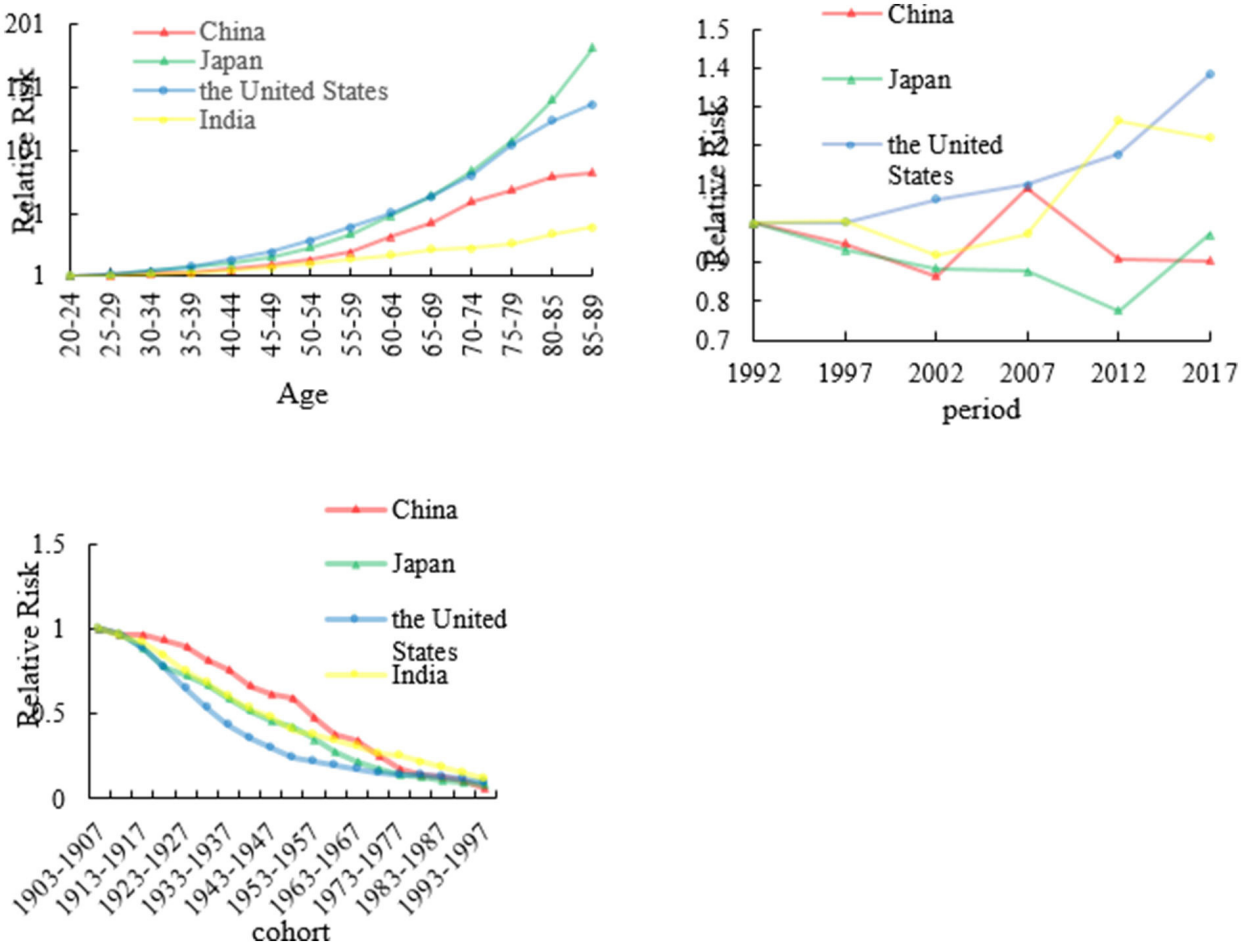
The age, period, and cohort effects on stomach cancer mortality across four countries.

We analyzed stomach RRs in China, Japan, the US, and India from 1992 to 2017 which are shown in Figure 4 and Appendix 3. The age effects for China, Japan, the US, and India showed that the death rate of stomach cancer had consistent increasing trends with age. In China and India, fluctuating period trends were presented in stomach cancer which showed a decreasing trend first during the period of 1992–2002 to 2007–2017; then, the period effects increased rapidly from 2002 to 2007. However, stomach cancer as a whole showed an upward change in the US. In Japan, the trend shows a decreasing trend first from 1992 to 2012 and then increased from 2012 to 2017. The cohort effect death rate patterns of stomach cancer in four countries showing a decreasing trend. The cohort effect of stomach cancer peaked from the years 1903 to 1907. The risk of stomach cancer-related death was the highest for those born in earlier birth cohort, but the later birth cohort of RRs was the lowest for four countries.

### Forecast of the mortality trends of three cancers using Holt model

**Figure 5:**
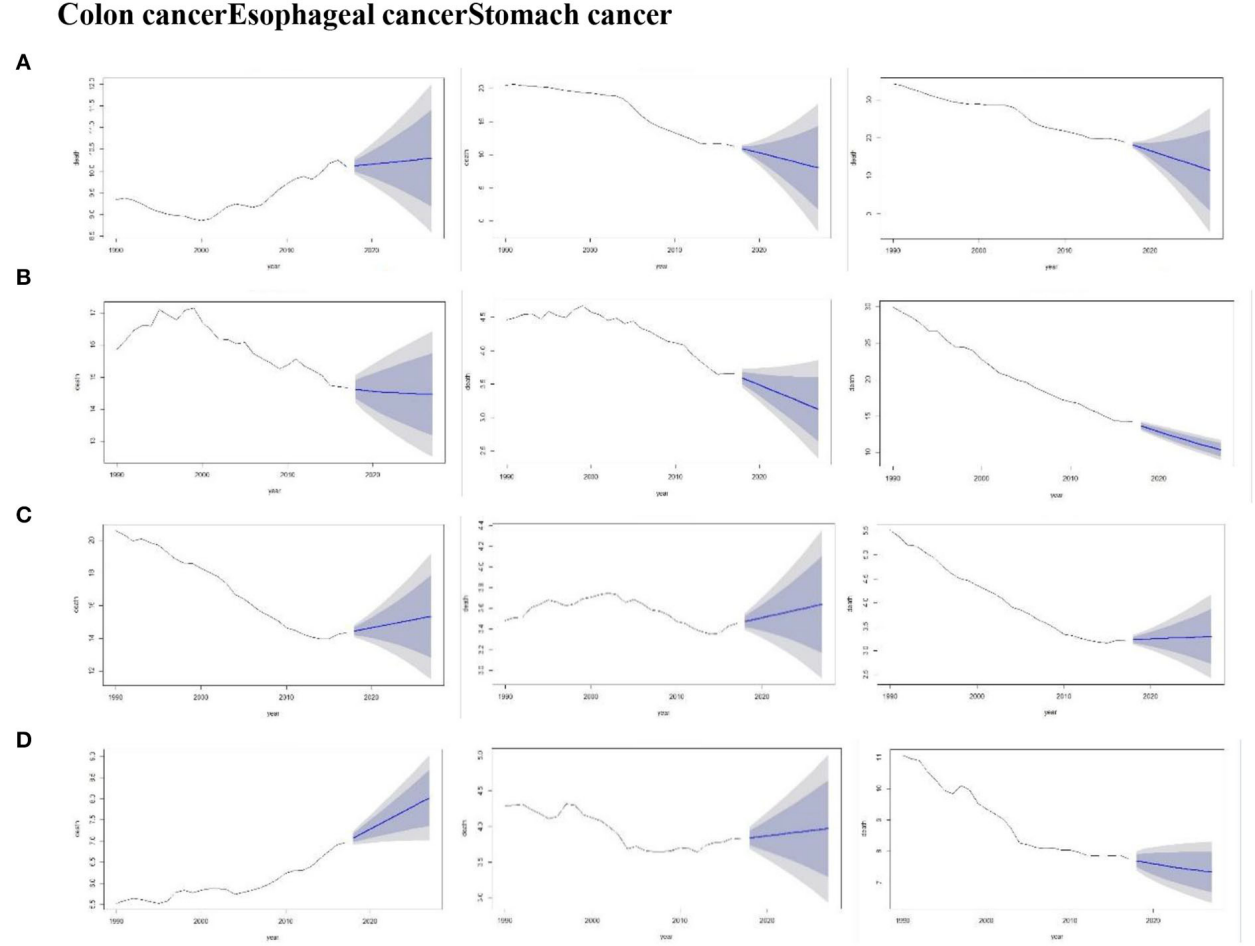
Forecast of the death rate using the Holt model for the period, 1990–2027 (Colon cancer, Esophageal cancer, and Stomach cancer) by China **(A)**, Japan **(B)**, the US **(C)**, and India **(D)**. Shades represent 80 and 95% prediction intervals.

Figure 5 depicts the forecasts of the three cancers by Holt models during the period 1990–2027 for four countries. The overall trend showed an increase in colon cancer trends for the period from 2018 to 2027 in China, the US, and India. Whereas the forecast trend of stomach cancer in China, Japan, and India showed a slightly declined trend. However, it was also observed that the confidence interval of colon cancer in China was wider than that in Japan. This indicated more uncertainty during that period.

## Discussion

The study of GBD 2017 data shows trends and patterns of change for three cancers in four countries. The study revealed an upward trend in the mortality rate of colon cancer. Colon cancer also shows a rapidly increasing trend in age effect values in all age groups. However, the age effect values for esophageal cancer in Japan differed significantly from those of the other three countries. The differences in the esophageal cancer and stomach cancer period effects across the countries were also significant. Age and birth year of cases and years or time during which they have lived are the three foremost time-reliant factors which are capable of independently impacting the time trends of cancer incidence and mortality. Therefore, APC model agrees with the identification problem. To overcome this problem and provide better insights into disease death rates, we have used the intrinsic estimator technique.

In China, gastric/stomach (23), colorectal (24), and esophageal (25) cancers ranked the second, third, and fifth among all the malignancies, respectively. Cancer statistics has shown roughly the diagnosis of 1.2 million new gastric cases all over the world and among which around 40% came from China representing its high incidence and mortality among the Chinese population (23). Colorectal cancer is also a leading cause of cancer-related deaths in China and the risk of the disease increases with age, particularly after 35 years of age and attains the peak among individuals aged 80–84 years (26). Esophageal cancer is the fourth leading cause of cancer-related mortality, its incidence is more common in rural areas, and among males with greater incidence above the age of forty years (25–27). We have found that death rate of all the three cancer types showed slightly increasing trends and declined at the end of the period possibly due to better treatment and healthcare facilities in China. Following the similar pattern of cancer incidence (23, 24), the ASMR of stomach cancer is significantly higher than that of colon cancer. Interestingly, the death rate of colon and esophageal cancer are nearly equal in 2017, representing a lesser rate of early cancer diagnosis and sub-standard treatment approaches offered in the diverse regions of China. The death rate of colon cancer peaks at the age of 80 to 90 years, highlighting age as a confounding factor in cancer incidence and mortality, which is consistent with the previously reported literature (28, 29). It has been suggested to offer regular colon cancer screening of people over 60 years of age who are identified as a high-risk population due to limited treatment-related resources in China (30).

Additionally, this death rate trend keeps on increasing with the time period of every year which might be due to the adoption of westernized lifestyle and less treatment facilities in some of the regions. As some of the regions in China have developed more in certain type of cancers due to their local environmental and lifestyle behaviors or traditions, elucidating the role these factors may help in controlling or decreasing the cancer burden (31). All the cancer types have shown an increasing trend with increasing age possibly due to environmental and genetic changes. Death rate of stomach cancer began to rise at the age of 35 years among the Chinese population. Increased prevalence of Helicobacter pylori, improper sanitation, and unhygienic storage of food and its consumption are the basic reasons behind the increased stomach cancer incidence and mortality in the younger population (32). It has been observed that stomach cancer trend first rises and then declines with the period showing betterment in the healthcare and treatment facilities. Consistent with the results of studies by other researchers, the uptrend in the ASMRs may be caused due to the period RRs of esophageal cancer and stomach cancer from 2004 to 2007 in China (33).

In Japan, the ASMR of colon cancer remains at the highest position followed by stomach and esophageal cancer mortality. Lifestyle and changes in dietary patterns, such as obesity in Japan since World War II, increased the uptake of alcohol and beverages with less to no physical activity (34). In Japan, the National Health and Nutrition Survey exposed a vibrant rise in the energy obtained from fat and showed that it rises from 7.0 to 26.6% in 1946 to 2000 indicating the establishment of westernized diet among the Japanese population with time (35). We have observed that Japan has rapid colon cancer death, peaking at the age of 75 to 80 years and this death rate increases with every calendar year. In 1992, Japan has introduced colorectal screening programs using immunochemical faucal occult blood testing for residents aged forty years or above to control the disease spread, and those who are tested positive on the initial screening are meant to opt for sigmoidoscopy or colonoscopy for confirmation (36). Besides all these preventive measures, it is still hard to control the disease spread which might be due to poor dietary patterns. Although the esophageal cancer is much lower than colon cancer in Japan, better socioeconomic conditions along with the introduction of screening programs might partially explain these results. One of the studies has analyzed the esophageal mortality trends from 1960–2000 in Japan and has observed a U-shaped curve among the age group of 45 to 79 years which is higher in males for mortality trends using the WHO data (37).

Although Japan has introduced screening programs for precursor lesions and early-stage detection in some populations, prevention persists to be the appropriate approach to lessen the esophageal cancer burden most probably by modifying the possible risk factors like smoking and alcohol consumption (7). The cohort studies showed that compared with people who never drink alcohol, people who drink 100 g of pure alcohol a day have 11.71 times the risk of esophageal cancer in Japan (38). In Japan, the ASMR of stomach cancer increases with the age particularly after the age of 40 years. While analyzing the period, a decreasing trend has been observed first from 1992 to 2012 and then it dramatically increased from 2012 to 2017. Whereas in the birth cohort, a declining trend was observed, and people born in later years have less death rate as compared to the one born in earlier times. One of the prospective cohort studies in Scotland established that men with adverse socioeconomic status in their childhood had greater stomach cancer mortality rates which is independent of their lifestyles in the adulthood (39). Researchers have thought that there might be two hypotheses for the mechanisms underlying this type of correlation including a greater biological vulnerability to ailment acquired unswervingly by poor resource settings in childhood and unhealthy lifestyles attained/activated through childhood environments and upheld throughout their adulthood. Poor dietary habits that do not meet the nutritional standards, smoking, and alcohol consumption are possible causes of stomach cancer and aforementioned mechanisms (40).

In the US, the ASMR of colon cancer remains peaking at the age of 70–80 years followed by stomach and esophageal cancer mortality rates. Interestingly, the death rate of colon cancer decreases over the course of period possibly due to better treatment facilities. Overall, this death rate is still much higher than the other selected countries like China and Japan. However, in the US, the death rate of esophageal cancer generally increased with age and period and significantly pronounced in earlier age groups. While stomach cancer rises in the earlier stages and then declines, t the death rate was higher among the elderly people. One of the probable justifications for the detected decrease in cohort effect, which is strictly linked to childhood socioeconomic status and strongly based on proof from epidemiological, biological, and clinical studies, is the declining prevalence of Helicobacter pylori infection in younger cohorts. Another potential reason for the decreasing cohort trend is possibly due to better health and environmental facilities (41, 42).

The ASMR of esophageal cancer in India is lower than that of stomach and colon cancer, which is concordant with the findings of another study from India highlighting the increasing colorectal cancer mortality rates (43). Death rate of all the cancer types increases with increased age in India; in the colorectal cancer, it peaks at the age group of 80 to 90 years. But this rate was lower than the US and Japan possibly due to less westernized diet in some of the regions in India. Death rate due to stomach and esophageal cancer starts at the age group of 35 years among the Indians. Poor and unhygienic feeding habits might be the reason behind the early onset of these cancers in India (44). Studies from India are limited but have shown certain reasons for stomach cancer development, such as Helicobacter pylori infection, pickled food, spicy food, smoking, and alcohol consumption (45).

As we all know, cancer burden has been alarming around the globe, magnified by aging process and increasing population density (46). Irregularities in various lifestyle-related factors like smoking, alcohol consumption, being obese/overweight, reduced physical activity, less intake of vegetable and fruits along with bacterial/viral infections can lead to carcinogenesis (47). However, it is not possible to quantify the impact of all the established risk factors due to data constraints (48). Some other factors are categorized as modifiable risk factors and are not decisively recognized yet such as the intake of processed or red meat, vegetables, fruits, dietary calcium, fiber, and vitamins (49).

## Limitation

First, the data collected in the present study was based on GBD 2017 and from data provided by local governments of worldwide countries, which may have very different systems for collecting vital statistics and methods for confirming the cause of death. In addition, although the IE method has unbiased and larger estimation, studies with more risk factors are needed to explain more accurate results in the future. However, GBD provides us with an important platform for international comparison.

## Conclusion

In conclusion, the ASMR of colon cancer ranked top among all the selected countries i.e., China, Japan, the US, and India. The trend of death rates among colon, stomach, and esophageal cancers has been increased in all countries with declining cohort trends. Excessive struggle is required not only to create public awareness and endorse early detection of cancer but also to deliver more manageable health services, adequate finance, and suitable cancer care-related infrastructure around the globe.

## Data availability statement

Publicly available datasets were analyzed in this study. This data can be found here: http://ghdx.healthdata.org/gbd-results-tool.

## Author contributions

YC designed this context, acquired data and analyzed and drafted the article. YY had access to the data and controlled the decision to publish. GC, GT and SH revised the article. All authors contributed to the article and approved the submitted version.

## Funding

This study was funded by the National Natural Science Foundations (NSFC) of China (grant number 81973153) and supported by the Fundamental Research Funds for the Central Universities of Central South University (grant number 2022ZZTS0845).

## Conflict of interest

The authors declare that the research was conducted in the absence of any commercial or financial relationships that could be construed as a potential conflict of interest.

## Publisher's note

All claims expressed in this article are solely those of the authors and do not necessarily represent those of their affiliated organizations, or those of the publisher, the editors and the reviewers. Any product that may be evaluated in this article, or claim that may be made by its manufacturer, is not guaranteed or endorsed by the publisher.

## Abbreviations

APC: age-period-cohort
ASMR: age-standardized mortality rate
DALYs: disability-adjusted life years
GBD: global burden of disease
IHME: Institute for Health Metrics and Evaluation
YLL: years of life lost
YLD: years lived with disability
AIC: Akaike information criteria
BIC: Bayes information criteria
AAPC: average annual percentage change.
