# Supplementary material for: Secular trends in the mortality of gastrointestinal cancers across China, Japan, the US, and India: An age-period-cohort, Joinpoint analyses, and Holt forecasts
Source: Front Public Health. 2022 Sep 29;10:925011. doi: 10.3389/fpubh.2022.925011 (PMC9559557; doi:10.3389/fpubh.2022.925011)
Supplement: Supplementary file 1 [file Data_Sheet_1.docx]

**Appendix 1.** Colon cancer death of 95% confidence interval (CI) due to age period cohort.

| Variables | Coef,95%CI | | | | |
| --- | --- | --- | --- | --- | --- |
|  | China Japan The U.S India | | | | |
| Age |  |  |  |  |  |
| 20-24 | -2.18(-3.34,-1.02) | -3.11(-4.74,-1.49) | -3.22(-4.71,-1.73) | | -2.52(-4.24,-0.79) |
| 25-29 | -1.71(-2.49,-0.93) | -2.49(-3.56,-1.42) | -2.52(-3.49,-1.56) | | -2.26(-3.57,-0.94) |
| 30-34 | -1.25(-1.86,-0.64) | -1.79(-2.56,-1.03) | -1.81(-2.51,-1.10) | | -1.56(-2.49,-0.63) |
| 35-39 | -1.20(-1.75,-0.65) | -1.27(-1.87,-0.67) | -1.22(-1.78,-0.67) | | -1.23(-2.01,-0.45) |
| 40-44 | -0.88(-1.32,-0.43) | -0.74(-1.22,-0.26) | -0.65(-1.10,-0.21) | | -0.76(-1.39,-0.13) |
| 45-49 | -0.53(-0.90,-0.16) | -0.23(-0.62,0.15) | -0.10(-0.47,0.26) | | -0.21(-0.71,0.29) |
| 50-54 | -0.23(-0.54,0.08) | 0.24(-0.07,0.55) | 0.35(0.06,0.65) | | 0.17(-0.24,0.59) |
| 55-59 | 0.06(-0.20,0.32) | 0.61(0.36,0.86) | 0.70(0.47,0.94) | | 0.44(0.10,0.78) |
| 60-64 | 0.50(0.29,0.70) | 0.92(0,71,1.12) | 1.00(0.81,1.19) | | 0.72(0.44,1.00) |
| 65-69 | 0.86(0.68,1.03) | 1.12(0.95,1.29) | 1.19(1.03,1.35) | | 0.95(0.71,1.18) |
| 70-74 | 1.24(1.08,1.40) | 1.33(1.16,1.49) | 1.35(1.20,1.50) | | 1.20(0.98,1.41) |
| 75-79 | 1.54(1.36,1.71) | 1.54(1.35,1.72) | 1.49(1.33,1.65) | | 1.44(1.21,1.67) |
| 80-85 | 1.81(1.60,2.01) | 1.80(1.57,2.03) | 1.66(1.47,1.85) | | 1.76(1.48,2.03) |
| 85-89 | 1.98(1.73,2.23) | 2.08(1.80,2.36) | 1.78(1.55,2.02) | | 1.85(1.52,2.19) |
| Period |  |  |  | |  |
| 1992 | -0.19(-0.36,-0.03) | -0.27(-0.45,-0.09) | -0.26(-0.42,-0.09) | | -0.34(-0.57,-0.11) |
| 1997 | -0.17(-0.29,-0.05) | -0.14(-0.26,-0.02) | -0.14(-0.25,-0.04) | | -0.25(-0.41,-0.09) |
| 2002 | -0.07(-0.16,0.02) | -0.05(0.12,0.02) | -0.03(-0.09,0.04) | | -0.09(-0.21,0.02) |
| 2007 | 0.03(-0.06,0.12) | 0.04(-0.03,0.12) | 0.02(-0.05,0.09) | | 0.11(0.00,0.22) |
| 2012 | 0.16(0.04,0.27) | 0.16(0.04,0.28) | 0.11(0.00,0.22) | | 0.11(-0.05,0.27) |
| 2017 | 0.24(0.09,0.40) | 0.26(0.08,0.43) | 0.29(0.14,0.45) | | 0.46(0.25,0.68) |
| Cohort |  |  |  | |  |
| 1903-1907 | 0.78(0.41,1.15) | 1.22(0.83,1.62) | 1.43(.07,1.79) | | 0.98(0.47,1.49) |
| 1908-1912 | 0.72(0.41,1.03) | 1.13(0.79,1.47) | 1.30(1.00,1.60) | | 0.94(0.52,1.36) |
| 1913-1917 | 0.70(0.43,0.97) | 1.01(0.72,1.31) | 1.15(0.89,1.41) | | 0.93(0.57,1.29) |
| 1918-1922 | 0.68(0.44,0.93) | 0.87(0.61,1.14) | 0.98(0.75,1.21) | | 0.87(0.56,1.19) |
| 1923-1927 | 0.66(0.42,0.89) | 0.77(0.53,1.02) | 0.79(0.58,1.01) | | 0.75(0.46,1.04) |
| 1928-1932 | 0.62(0.39,0.84) | 0.67(0.43,0.91) | 0.59(0.38,0.80) | | 0.64(0.36,0.93) |
| 1933-1937 | 0.56(0.31,0.81) | 0.52(0.25,0.78) | 0.38(0.15,0.61) | | 0.52(0.21,0.84) |
| 1938-1942 | 0.45(0.16,0.74) | 0.36(0.06,0.66) | 0.18(-0.09,0.44) | | 0.35(-0.02,0.71) |
| 1943-1947 | 0.39(0.06,0,72) | 0.23(-0.11,0.58) | -0.03(-0.33,0.28) | | 0.24(-0.18,0.66) |
| 1948-1952 | 0.42(0.05,0.80) | 0.11(-0.30,0,51) | -0.20(-0.56,0.16) | | 0.05(-0.44,0.55) |
| 1953-1957 | 0.27(-0.16,0.71) | -0.06(-0.52,0.40) | -0.31(-0.72,0.10) | | -0.06(-0.63,0.51) |
| 1958-1962 | 0.02(-0.48,0.52) | -0.26(-0.79,0.26) | -0.42(-0.89,0.05) | | -0.16(-0.81,0.49) |
| 1963-1967 | 0.00(-0.55,0.55) | -0.46(-1.06,0.15) | -0.54(-1.07,0.00) | | -0.29(-1.02,0.45) |
| 1968-1972 | -0.29(-0.92,0.34) | -0.62(-1.32,0.08) | -0.67(1.29,-0.05) | | -0.49(-1.34,0.36) |
| 1973-1977 | -0.68(-1.48,0.11) | -0.77(-1.62,0.07) | -0.75(-1.50,-0.01) | | -0.59(-1.61,0.43) |
| 1978-1982 | -0.98(-1.99,0.03) | -0.94(-2.02,0.13) | -0.79(-1.71,0.13) | | -0.79(-2.07,0.49) |
| 1983-1987 | -1.16(2.41,0.10) | -1.11(-2.58,0.36) | -0.87(-2.10,0.36) | | -0.99(-2.67,0.69) |
| 1988-1992 | -1.35(-3.17,0.47) | -1.26(-3.51,0.99) | -1.01(-2.90,0.87) | | -1.23(-3.83,1.37) |
| 1993-1997 | -1.82(-5.84,2.19) | -1.42(-5.94,3.10) | -1.21(-5.09,2.68) | | -1.67(-6,73,3.38) |
| AIC | 5.20 | 5.43 | 5.52 | | 4.73 |
| BIC | -208.27 | -211.71 | -212.09 | | -209.76 |
| Deviance | 4.41 | 0.96 | 0.59 | | 2.92 |

Note: AIC, Akaike’s information criterion; BIC, Bayesian information criterion.

**Appendix 2.** Esophageal cancer death of 95% confidence interval (CI) due to age period cohort.

| Variables | Coef,95%CI | | | | |
| --- | --- | --- | --- | --- | --- |
|  | China Japan The U.S India | | | | |
| Age |  |  |  |  |  |
| 20-24 | -3.08(-5.10,-1.06) | -3.94(11.03,3.06) | -3.55(-8.12,1.03) | | -2.55(-4.56,-0.54) |
| 25-29 | -2.87(-4.42,-1.33) | -3.61(-8.85,1.63) | -2.89(-5.84,0.07) | | -2.27(-3.82,-0.72) |
| 30-34 | -2.24(-3.33,-1.14) | -2.87(-6.48,0.73) | -2.17(-4.27,-0.06) | | -1.83(-3.07,-0.60) |
| 35-39 | -1.75(-2.59,-0.92) | -1.90(-4.34,0.53) | -1.34(-2.87,0.19) | | -1.25(-2.19,-0.30) |
| 40-44 | -0.75(-1.34,-0.17) | -0.74(-2.50,1.02) | -0.49(-1.66,0.67) | | -0.55(-1.27,0.17) |
| 45-49 | -0.17(-0.63,0.29) | 0.19(-1.21,1.58) | 0.18(-0.76,1.12) | | 0.05(-0.52,0.62) |
| 50-54 | 0.29(-0.08,0.65) | 0.90(-0.22,2.02) | 0.71(-0.05,1.47) | | 0.51(0.05,0.98) |
| 55-59 | 0.65(0.36,0.94) | 1.38(0.50,2.27) | 1.10(0.49,1.71) | | 0.79(0.41,1.17) |
| 60-64 | 1.15(0.92,1.38) | 1.66(0.96,2.37) | 1.32(0.84,1.80) | | 0.91(0.59,1.23) |
| 65-69 | 1.44(1.22,1.66) | 1.79(1.18,2.40) | 1.43(1.03,1.83) | | 1.02(0.75,1.30) |
| 70-74 | 1.72(1.46,1.97) | 1.83(1.19,2.47) | 1.48(1.09,1.86) | | 1.13(0.87,1.39) |
| 75-79 | 1.81(1.49,2.13) | 1.81(1.03,2.60) | 1.45(1.00,1.89) | | 1.30(1.01,1.58) |
| 80-84 | 1.93(1.53,2.32) | 1.78(0.79,2,77) | 1.42(0.87,1.96) | | 1.32(0.98,1.67) |
| 85-89 | 1.90(1.41,2.39) | 1.72(0.49,2.95) | 1.35(0.67,2.03) | | 1.41(0.99,1.83) |
| Period |  |  |  | |  |
| 1992 | -0.01(-0.27,0.25) | -0.35(-1.07,0.36) | -0.41(-0.85,0.04) | | -0.22(-0.49,0.06) |
| 1997 | 0.03(-0.13,0.19) | -0.20(-0.64,0.24) | -0.23(-0.52,0.06) | | -0.11(-0.31,0.08) |
| 2002 | -0.10(-0.19,-0.02) | -0.04(-0.23,0.14) | -0.04(-0.20,0.12) | | -0.14(-0.28,0.01) |
| 2007 | 0.20(0.12,0.28) | 0.09(-0.10,0.29) | 0.08(-0.09,0.25) | | -0.02(-0.17,0.12) |
| 2012 | -0.07(-0.24,0.09) | 0.14(-0.31,0.58) | 0.21(-0.08,0.50) | | 0.20(0.00,0.39) |
| 2017 | -0.04(-0.29,0.22) | 0.37(-0.35,1.08) | 0.39(-0.04,0.82) | | 0.29(0.03,0.56) |
| Cohort |  |  |  | |  |
| 1903-1907 | 1.14(0.50,1.77) | 1.79(0.14,3.45) | 1.26(0.22,2.31) | | 1.05(0.43,1.68) |
| 1908-1912 | 1.09(0.53,1.65) | 1.53(0.11,2.95) | 1.18(0.29,2.06) | | 1.00(0.49,1.51) |
| 1913-1917 | 1.08(0.58,1.59) | 1.29(0.06,2.52) | 1.11(0.34,1.87) | | 0.93(0.49,1.37) |
| 1918-1922 | 1.04(0.58,1.50) | 1.05(-0.03,2.12) | 1.02(0.35,1.69) | | 0.81(0.43,1.20) |
| 1923-1927 | 0.96(0.52,1.40) | 0.93(-0.05,1.92) | 0.90(0.29,1.52) | | 0.69(0.34,1.20) |
| 1928-1932 | 0.85(0.40,1.30) | 0.82(-0.15,1.79) | 0.75(0.14,1.35) | | 0.58(0.23,0.92) |
| 1933-1937 | 0.72(0.25,1.19) | 0.66(-0.38,1.70) | 0.57(-0.08,1.23) | | 0.45(0.07,0.83) |
| 1938-1942 | 0.57(0.05,1.09) | 0.51(-0.67,1.68) | 0.39(-0.35,1.13) | | 0.28(-0.15,0.72) |
| 1943-1947 | 0.50(-0.08,1.08) | 0.35(-1.01,1.71) | 0.21(-0.64,1.05) | | 0.18(-0.33,0.68) |
| 1948-1952 | 0.42(-0.22,1.07) | 0.15(-1.42,1.73) | 0.02(-0.96,1.00) | | -0.01(-0.60,0.58) |
| 1953-1957 | 0.21(-0.52,0.94) | -0.10(1.91,1.71) | -0.15(-1.27,0.97) | | -0.11(-0.78,0.56) |
| 1958-1962 | -0.09(-0.92,0.74) | -0.37(-2.44,1.70) | -0.34(-1.62,0.94) | | -020(-0.97,0.56) |
| 1963-1967 | -0.28(-1.21,0.65) | -0.64(-3.00,1.72) | -0.57(-2.03,0.89) | | -0.34(1.21,0.53) |
| 1968-1972 | -0.66(-1.74,0.41) | -0.91(-3.63,1.81) | -0.80(-2.50,0.89) | | -0.50(-1.50,0.51) |
| 1973-1977 | -1.05(-2.43,0.33) | -1.07(-4.38,2.23) | -0.93(-2.97,1.12) | | -0.59(-1.82,0.64) |
| 1978-1982 | -1.24(-3.14,0.67) | -1.22(-5.75,3.32) | -0.97(-3.59,1.64) | | -0.73(-2.34,0.87) |
| 1983-1987 | -1.39(-3.93,1.14) | -1.43(-8.33,5.46) | -1.05(-4.70,2.60) | | -0.88(-3.02,1.26) |
| 1988-1992 | -1.71(-5.81,2.39) | -1.58(12.02,8.86) | -1.18(-6.75,4.40) | | -1.16(-4.27,1.95) |
| 1993-1997 | -2.16(-10.55,6.23) | -1.75(20.62,17.12) | -1.42(13.03,10.19) | | -1.45(7.42,4.51) |
| AIC | 5.39 | 4.08 | 4.10 | | 4.36 |
| BIC | -202.10 | -211.84 | -212.52 | | -212.00 |
| Deviance | 10.57 | 0.84 | 0.16 | | 0.67 |

**Appendix 3.** Stomach cancer death of 95% confidence interval (CI) due to age period cohort.

| Variables | Coef,95%CI | | | | |
| --- | --- | --- | --- | --- | --- |
|  | China Japan The U.S India | | | | |
| Age |  |  |  |  |  |
| 20-24 | -2.67(-3.65,1.70) | -3.12(-4.53,-1.72) | -3.16(-5.92,-0.40) | | -2.20(-3.17,-1.23) |
| 25-29 | -2.06(-2.69,1.43) | -2.22(-3.04,-1.41) | -2.26(-3.90,-0.62) | | -1.93(-2.70,-1.17) |
| 30-34 | -1.49(-1.96,-1.02) | -1.60(-2.20,-1.00) | -1.56(-2.77,-0.34) | | -1.41(-2.00,-0.82) |
| 35-39 | -1.30(-1.71,-0.90) | -1.10(-1.57,-0.62) | -1.01(-1.98,-0.04) | | -0.96(-1.45,-0.48) |
| 40-44 | -0.78(-1.09,-0.47) | -0.72(-1.10,-0.33) | -0.54(-1.35,0.26) | | -0.43(-0.81,-0.04) |
| 45-49 | -0.38(-0.63,-0.12) | -0.34(-0.66,-0.02) | -0.15(-0.82,0.52) | | -0.18(-0.51,0.15) |
| 50-54 | -0.01(-0.22,0.20) | 0.04(-0.22,0.30) | 0.22(-0.33,0.77) | | 0.18(-0.10,0.45) |
| 55-59 | 0.32(0.16,0.49) | 0.42(0.22,0.63) | 0.52(0.07,0.97) | | 0.47(0.25,0.70) |
| 60-64 | 0.81(0.67,0.94) | 0.77(0.60,0.94) | 0.78(0.42,1.15) | | 0.69(0.50,0.88) |
| 65-69 | 1.11(0.99,1.23) | 1.06(0.91,1.20) | 1.00(0.70,1.30) | | 0.89(0.73,1.06) |
| 70-74 | 1.43(1.31,1.55) | 1.32(1.18,1.47) | 1.24(0.97,1.51) | | 0.95(0.80,1.11) |
| 75-79 | 1.57(1.43,1.71) | 1.57(1.40,1.73) | 1.50(1.21,1.79) | | 1.10(0.94,1.27) |
| 80-85 | 1.71(1.54,1.88) | 1.83(1.63,2.03) | 1.66(1.32,2.01) | | 1.34(1.16,1.52) |
| 85-89 | 1.75(1.54,1.96) | 2.09(1.84,2.33) | 1.77(1.34,2.19) | | 1.48(1.26,1.70) |
| Period |  |  |  | |  |
| 1992 | 0.05(-0.07,0.17) | 0.10(-0.04,0.25) | -0.11(-0.40,0.18) | | -0.06(-0.21,0.10) |
| 1997 | 0.00(-0.08,0.08) | 0.03(-0.07,0.13) | -0.11(-0.30,0.09) | | -0.05(-0.16,0.06) |
| 2002 | -0.10(-0.15,-0.04) | -0.02(-0.08,0.04) | -0.05(-0.18,0.09) | | -0.14(-0.23,-0.04) |
| 2007 | 0.14(0.08,0.19) | -0.03(-0.10,0.03) | -0.01(-0.16,0.13) | | -0.08(-0.18,0.02) |
| 2012 | -0.04(-0.13,0.04) | -0.15(-0.26,-0.05) | 0.06(-0.15,0.25) | | 0.18(0.07,0.29) |
| 2017 | -0.05(-0.17,0.07) | 0.07(-0.07,0.22) | 0.22(-0.07,0.50) | | 0.14(0.00,0.29) |
| Cohort |  |  |  | |  |
| 1903-1907 | 0.90(0.60,1.20) | 1.08(0.75,1.42) | 1.22(0.57,1.87) | | 0.89(0.56,1.23) |
| 1908-1912 | 0.86(0.60,1.13) | 1.05(0.75,1.34) | 1.19(0.64,1.74) | | 0.85(0.58,1.12) |
| 1913-1917 | 0.86(0.62,1.10) | 0.96(0.71,1.22) | 1.11(0.63,1.58) | | 0.81(0.58,1.05) |
| 1918-1922 | 0.84(0.62,1.06) | 0.84(0.60,1.07) | 0.96(0.53,1.39) | | 0.72(0.51,0.93) |
| 1923-1927 | 0.79(0.57,1.00) | 0.77(0.54,0.99) | 0.79(0.39,1.19) | | 0.61(0.41,0.80) |
| 1928-1932 | 0.70(0.49,0.92) | 0.69(0.46,0.92) | 0.59(0.19,0.99) | | 0.51(0.32,0.70) |
| 1933-1937 | 0.63(0.40,0.86) | 0.57(0.33,0.82) | 0.39(-0.05,0.83) | | 0.39(0.18,0.61) |
| 1938-1942 | 0.49(0.24,0.74) | 0.43(0.15,0.70) | 0.19(-0.31,0.69) | | 0.26(0.01,0.50) |
| 1943-1947 | 0.42(0.14,0.70) | 0.31(0.00,0.62) | 0.00(-0.58,0.58) | | 0.16(-0.12,0.44) |
| 1948-1952 | 0.38(0.06,0.69) | 0.21(-0.14,0.57) | -0.18(-0.85,0.50) | | -0.01(-0.34,0.32) |
| 1953-1957 | 0.18(-0.18,0.53) | 0.04(-0.37,0.44) | -0.29(-1.06,0.48) | | -0.09(-0.45,0.29) |
| 1958-1962 | -0.07(-0.48,0.33) | -0.20(-0.66,0.27) | -0.40(-1.27,0.48) | | -0.17(0.60,0.25) |
| 1963-1967 | -0.17(-0.62,0.28) | -0.44(-0.97,0.10) | -0.53(-1.53,0.46) | | -0.28(-0.77,0.20) |
| 1968-1972 | -0.47(-0.99,0.04) | -0.68(-1.30,-0.05) | -0.67(-1.82,0.47) | | -0.44(-0.99,0.11) |
| 1973-1977 | -0.85(-1.51,-0.19) | -0.86(-1.62,-0.10) | -0.74(-2.09,0.61) | | -0.49(-1.13,0.15) |
| 1978-1982 | -1.07(-1.92,-0.21) | -1.00(-1.96,-0.04) | -0.76(-2.40,0.88) | | -0.64(1.47,0.18) |
| 1983-1987 | -1.18(-2.26,-0.10) | -1.13(-2.44,0.18) | -0.81(-2.97,1.35) | | -0.77(-1.84,0.30) |
| 1988-1992 | -1.36(-3.02,0.29) | -1.27(-3.26,0.73) | -0.93(-4.26,2.39) | | -1.01(-2.57,0.55) |
| 1993-1997 | -1.88(-5.85,2.09) | -1.38(-5.62,2.86) | -1.11(-8.45,6.22) | | -1.29(-4.34,1.75) |
| AIC | 6.19 | 5.70 | 4.10 | | 5.25 |
| BIC | -193.46 | -210.12 | -212.52 | | -210.85 |
| Deviance | 19.22 | 2.56 | 0.16 | | 1.83 |
